# Enhancement of vascular visualization in laser speckle contrast imaging based on image algorithms

**DOI:** 10.1117/1.JBO.30.5.056010

**Published:** 2025-05-29

**Authors:** Long Yan, Gongzhi Du, Xiaozheng Huang, Yiheng Xiao, Jinhua Bian, Yuanzhi Zhang, Huayi Hou, Min Min, Xiangbai Chen

**Affiliations:** a Wuhan Institute of Technology, Hubei Key Laboratory of Optical Information and Pattern Recognition, Wuhan, China; b Chinese Academy of Sciences, Anhui Institute of Optics and Fine Mechanics, Hefei Institutes of Physical Science, Hefei, China; c Tongji Hospital, Huazhong University of Science and Technology, Tongji Medical College, Department of Nursing, Wuhan, China

**Keywords:** laser speckle contrast imaging, image registration, image denoising, multi-focus image fusion, blood flow velocity

## Abstract

**Significance:**

In practical biomedical applications, obtaining clear and focused speckle images through laser speckle contrast imaging (LSCI) presents significant challenges. These challenges are often compounded by motion artifacts and image noise, which can adversely affect the effectiveness of vascular visualization in LSCI.

**Aim:**

We improved the visualization of blood flow in LSCI by focusing on three aspects: image registration, image denoising, and multi-focus image fusion.

**Approach:**

We employed the Lucas–Kanade (LK) optical flow pyramid method alongside block matching and three-dimensional filtering (BM3D) algorithm based on guided filtering with total variation regularization to effectively mitigate motion artifacts and noise. Furthermore, we proposed a multi-focus image fusion technique based on the multi-scale image contrast amplification (MUSICA) algorithm, aimed at enhancing high-frequency signals and minimizing the effects of defocusing in LSCI.

**Results:**

The LK optical flow registration algorithm demonstrates improvements in both average peak signal-to-noise ratio and imaging quality compared with non-registration methods. The improved BM3D method outperforms classical denoising algorithms in various image evaluation parameters within LSCI. In the case of using the multi-focus image fusion method based on the MUSICA method, the image quality assessment of the sum of modulus of gray difference squared showed an improvement of nearly six times compared with the defocused images without the use of the MUSICA method.

**Conclusions:**

Improvements in image processing algorithms, specifically in the areas of registration, denoising, and multi-focus image fusion, have significantly enhanced the visualization of blood flow in the vessels during practical applications of LSCI.

## 1 Introduction

Laser speckle contrast imaging (LSCI) is a real-time imaging technology used to monitor blood flow within biological tissues.^1^ LSCI leverages the characteristics of speckle patterns produced by laser light scattering on an object. These patterns are captured by a camera to yield information about the imaged area, thereby enabling the quantitative measurement of blood flow.^2^ LSCI presents several advantages, including full-field imaging, non-contact operation, and high spatiotemporal resolution. Consequently, it has been extensively utilized in medical fields such as dermatology, neurology, and ophthalmology for blood flow imaging.^3^^,^^4^ In traditional LSCI, the visualization of cerebral blood flow is influenced by several factors. Notably, the subject’s respiratory movements can introduce motion artifacts into the images captured by the camera. The artifact results in blurred vascular imaging and adversely affects speckle contrast analysis.^5^ In addition, the presence of noise can significantly degrade the signal-to-noise ratio of images. Sources of noise include static scattered light from surrounding tissues in biological systems, as well as interference from equipment such as light sources and cameras, which can introduce system noise, scattering noise, and statistical noise. In addition, the curvature of certain physiological structures can lead to defocusing during image acquisition, resulting in errors when estimating blood flow velocity. In practical applications, it is essential to implement supplementary measures to mitigate the impact of motion artifacts, image noise, and defocusing effects.

To maintain the position of the experimental subject consistently, it is essential to anesthetize and secure the subject prior to the commencement of imaging. In addition, mitigating motion artifact effects is crucial. Image registration serves as an effective technique for addressing and correcting motion artifacts. Miao et al.^6^ proposed a method based on registered laser speckle contrast analysis to improve the spatial resolution of cerebral blood flow imaging. Guilbert and Desjardins^7^ introduced an image decomposition–based method for correcting motion artifacts. Kim et al.^8^ proposed a method using sample entropy to replace standard deviation for contrast calculation to monitor blood flow changes and eliminate artifacts. In practical clinical applications, Chizari et al.^9^ manually extracted marked points and maximized the enhanced correlation coefficient between two frames to achieve frame alignment, thereby verifying the feasibility of using handheld LSCI to address motion artifacts in the treatment of psoriasis lesions. However, this method requires manual labeling and has some limitations when dealing with non-rigid transformation. In this study, we employed the Lucas–Kanade (LK) optical flow pyramid algorithm to track motion by directly using natural features such as textures and edges to mark corner points. It allows for non-rigid registration that accommodates not only translation, rotation, and scaling deformations but also elastic deformations using a thin-plate spline (TPS) coordinate transformation model. In LSCI, speckle features can be used to calculate the optical flow information of object motion, offering better adaptability to local deformations caused by breathing and heartbeat. LK is applicable in fields such as three-dimensional (3D) reconstruction, motion capture, and motion detection.^10^^,^^11^

In some studies on image denoising for LSCI, Song et al.^12^ proposed an image denoising algorithm based on an anisotropic diffusion filter (ADF) to enhance the signal-to-noise ratio. Among traditional image-denoising algorithms, block-matching and 3D filtering (BM3D) currently exhibits better filtering performance.^13^^–^^15^ However, for speckle noise in LSCI, the performance of the original BM3D algorithm does not meet the standard. Therefore, an improved version of the BM3D algorithm has been developed specifically for LSCI. Fu et al.^16^ introduced a fusion denoising method based on homomorphic transformation and 3D transform domain collaborative filtering. Cheng et al.^17^ proposed an adaptive BM3D algorithm based on Manhattan distance to address noise in LSCI. However, the Wiener filtering used in the final estimation stage of BM3D is a linear filter, which can cause damage to the edges of speckle images.^18^ This paper replaces Wiener filtering with guided filtering to preserve vascular edge details as much as possible, optimizing the relationship between the guide image and the input image through total variation (TV) regularization term.

The phenomenon of misfocus generally arises from insufficient depth of field in imaging cameras. Some researchers aim to achieve autofocus by extending the depth of field. Sigal et al.^19^ employed wavefront coding technology to extend the depth of field in LSCI systems. Ringuette et al.^20^ demonstrated an autofocus system based on a novel defocus measurement method for estimating blood flow velocity. Extending the depth of field typically requires hardware adjustments, but multi-focus image fusion can reduce misfocus effects without altering hardware equipment.^21^^,^^22^ Currently, related algorithms for multi-focus image fusion are continually evolving, achieving outstanding fused images by better-decomposing image information.^23^^–^^25^ However, there has been limited focus on image contrast during the multi-focus image fusion process, which is crucial for displaying vascular contrast in LSCI. This paper proposes a multi-focus image fusion method based on a multi-scale image contrast enhancement algorithm (MUSICA), utilizing image features during the fusion process to further enhance contrast in blood flow estimation. The MUSICA algorithm decomposes the image into a weighted sum of smooth, localized two-dimensional basis functions at multiple scales, by non-linearly amplifying the transform coefficients, to enhance the detail contrast.^26^

This study advances the visualization of blood flow in LSCI through three key improvements: image registration, noise reduction, and defocus correction. The registration method employs the LK optical flow pyramid algorithm, with TPS used as the coordinate transformation model. This approach effectively suppresses motion artifacts compared with unregistered images. In addition, the study introduces an innovative integration of the BM3D algorithm, improved by guided filtering and total variation regularization, innovatively applied to LSCI. This approach demonstrates superior performance compared with the original BM3D and several other denoising algorithms in LSCI applications. Furthermore, by fusing images from multiple focal planes and employing the MUSICA algorithm to enhance image contrast, the study addresses the defocus issue in LSCI and significantly improves the visualization of blood flow.

## 2 Theory

### 2.1 Calculation of Speckle Contrast

In LSCI, the velocity of scattering particles is closely related to the speckle contrast K, which can be expressed as

$$K=\frac{\sigma }{\overset{¯}{I}},$$ (1)

where σ represents the standard deviation of the light intensity of all pixels in the original speckle image, and $\overset{¯}{I}$ is the average light intensity of all pixels in the entire speckle image.^27^

Based on the exposure time T and the electric field decorrelation time $\tau_{c}$, speckle contrast K can be expressed as^28^

$$K=\sqrt{\frac{e^{-2T/\tau_{c}}-1+2(T/\tau_{c})}{2(T/\tau_{c}{)}^{2}}}.$$ (2)

Electric field decorrelation time $\tau_{c}$ reflects the rate of fluctuation of the electric field intensity, which is inversely proportional to the velocity of the scattering particles

$$\tau_{c}\propto \frac{1}{v}.$$ (3)

Therefore, the velocity of the scattering particles is inversely proportional to the blood flow velocity in the vascular section and the speckle contrast K

$$\frac{1}{K^{2}}\propto v.$$ (4)

To calculate the contrast K of speckle images, traditional LSCI techniques are categorized into three methods: (1) spatial speckle contrast imaging (sLSCI), (2) temporal speckle contrast imaging (tLSCI), and (3) spatio-temporal speckle contrast imaging (stLSCI).^29^ The spatial resolution of tLSCI is superior to that of sLSCI and stLSCI. In this paper, we employ the tLSCI method to calculate speckle contrast, with a sampling frame count of 50 frames.

### 2.2 Image Registration Based on the LK Optical Flow Pyramid Algorithm

LK uses the first frame in a continuous time series as the reference image, and subsequent frames are treated as floating images for registration. The Shi–Tomasi corner point detection method, employed in optical flow tracking, effectively mitigates the effects of lighting variations and noise in speckle experiments, demonstrating robustness.

Assuming that a camera captures images of an object within a short time frame, a point on the initial image M undergoes motion during this interval. It is necessary to find the corresponding point on the second image N and calculate the optical flow d that has occurred during this time. This is done by minimizing the error within the neighborhood constrained by the optical flow

$$\varepsilon (d)=\varepsilon (d_{x},d_{y})=\overset{u_{x}+\omega_{x}}{\underset{x=u_{x}-\omega_{x}}{\sum }}\overset{u_{y}+\omega_{y}}{\underset{y=u_{y}-\omega_{y}}{\sum }}(M(x,y)-N(x+d_{x},y+d_{y}){)}^{2},$$ (5)

where $\left(d_{x},d_{y}\right)$ represents the flow of light that has passed through. Within the neighborhood range $\left(\omega_{x},\omega_{y}\right)$, we set the integration window size to $\left(2\omega_{x}+M)\times 2(\omega_{y}+M\right)$. By introducing an image pyramid and determining the size of the integration window to balance the accuracy and robustness of the LK optical flow algorithm, the optical flow is iteratively calculated to optimize the tracking effect. The LK algorithm uses the optical flow tracking information from the current frame to calculate the initial tracking points for the next frame.

Using optical flow tracking, corresponding points between the current frame and the previous frame are matched to form paired point sets. However, the paired points generated by optical flow tracking often contain outliers. This study employs the random sample consensus (RANSAC) algorithm to filter out these erroneous matching point pairs. RANSAC achieves this by iterative random sampling and model validation, selecting inliers that fit the model. The algorithm randomly selects s pairs of points from the dataset for model estimation and calculates the reprojection error of all point pairs under the model, including data with errors below a certain threshold into the inlier set. The selection of the threshold can be manually configured within the algorithm parameters. Through iterative processes of resampling, model estimation, and inlier selection, the model with the maximum number of inliers is retained. The number of iterations k can be expressed as

$$k\geq \frac{\log(1-p)}{\log(1-(1-\varepsilon {)}^{s})},$$ (6)

where p represents the confidence level that is typically set at 0.995. ε represents the estimated proportion of outliers. The RANSAC algorithm typically employs the homography matrix as a model to compute the projection error. It evaluates the validity of a pair of matching points by comparing the projection error to a predefined threshold. If the error is below the threshold, the pair is deemed a valid match; otherwise, it is classified as an outlier.

The motion of the experimental object in LSCI is characterized by diverse movements, including overall translations, scaling, and other motions, as well as potential local movements. To accommodate this complexity, the coordinate transformation model employs the TPS model.^30^ TPS is a non-rigid transformation model that excels in correcting both local and global motion deviations. Its interpolation function is

$$f(x,y)=a_{1}+a_{2}x+a_{3}y+\overset{n}{\underset{i=1}{\sum }}w_{i}U(|p_{i}(x_{i},y_{i})-p(x,y)|),$$ (7)

where U is the basis function. Using the basis function and Eq. (7) along with matrix calculations, $a_{1},a_{2},a_{3}$, and $w_{i}$ are computed to solve for the function f. After interpolating the points on the image and applying correction, the mapped coordinates are obtained, achieving registration.

### 2.3 Image Denoising Based on Improved BM3D

**Fig. 1:**
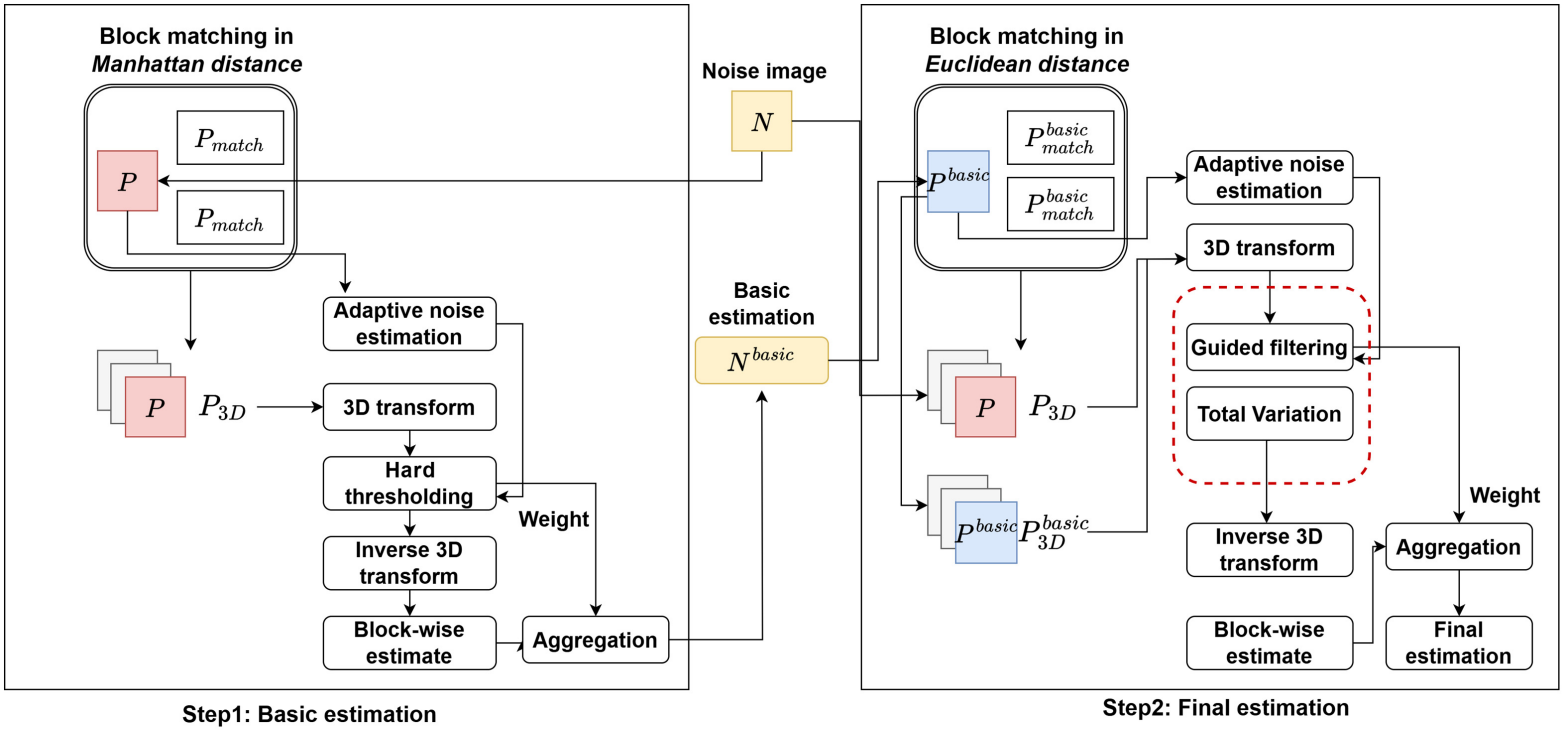
Schematic of addressing non-uniform noise in tLSCI by improved BM3D algorithm.

Speckle noise in images is typically non-uniform, and although the BM3D algorithm is highly effective for image denoising, its performance can degrade when dealing with non-uniform noise. The use of a Wiener filter in the final estimation step may lead to the blurring of image edges. To address this issue, guided filtering with total variation regularization can be employed as an alternative to the Wiener filter in the collaborative filtering process. This approach helps preserve edge details while effectively denoising the image. Figure 1 displays the schematic of addressing non-uniform noise in tLSCI by an improved BM3D algorithm.

In basic estimations, substituting the Euclidean distance with the Manhattan distance effectively mitigates the impact of non-uniform noise.^17^ However, in the low-frequency region, simple hard-threshold operations still fail to remove residual noise and may also lead to the loss of certain image details. This can significantly affect the analysis of blood flow information in LSCI. The image details lost due to Wiener filtering can be restored using guided filtering.^31^

Guided filtering is typically based on two images: the input image p and the guidance image I. Through these two images, the edges of the image can be effectively preserved

$$\left\{ \begin{array}{l} q_{i}=a_{k}I_{i}+b_{k},\;\forall \;i\in \omega_{k},\\ q_{i}=p_{i}-n_{i} \end{array},\right.$$ (8)

where p represents the input image, I denotes the guide image, q refers to the output image, and i and j are the pixel indices. It is assumed that there exists a local linear relationship between the output image and the guide image within a local window. k is the midpoint of the localized window, and n represents noise or texture.

By employing coefficients $a_{k}$ and $b_{k}$ to facilitate the calculations, the disparity between p and q is minimized. This approach ensures the preservation of a locally linear model, thereby enhancing the precision of the image transformation

$$E(a_{k},b_{k})=\sum_{i\in \omega_{k}}((a_{k}I_{i}+b_{k}-p_{i}{)}^{2}+\varepsilon a_{k}^{2}),$$ (9)

where ε is the regularization parameter; it prevents $a_{k}$ to become excessively large. However, during the denoising process of speckle images, the guiding image inevitably contains noise, which affects the performance of the guided filtering. Noise also introduces additional image gradients, and it is generally believed that image gradients are limited. Therefore, the problem of image denoising needs to be transformed into a gradient optimization problem. The norm of the image gradient is also known as total variation.^32^ Mathematically, total variation is defined as

$$V_{\mathrm{TV}}(p)=\sum_{i,j}|p_{i+1,j}-y_{i,j}|+|(p_{i,j+1}-p_{i,j})|.$$ (10)

In the guided image, total variation regularization is used to optimize noisy speckle images. The key to this optimization lies in $a_{k}$, as $a_{k}$ is the decisive factor in gradient calculation for guided filtering. Therefore, two parameters, $a_{k}$ and $b_{k}$, can be determine

$$\left\{ \begin{array}{l} a_{k}=\frac{\frac{1}{\left|\omega \right|}\underset{i\in \omega_{k}}{\sum }I_{i}p_{i}-\mu_{k}{\overset{¯}{p}}_{k}}{\sigma_{k}^{2}+\varepsilon }+\lambda V_{\mathrm{TV}}(I),\\ b_{k}={\overset{¯}{p}}_{k}-a_{k}\mu_{k},\\ \lambda =\lambda_{\mathrm{TV}}/I_{\text{size}} \end{array}\right.$$ (11)

where λ is the smoothing factor of the TV regularization term, and $\lambda_{\mathrm{TV}}$ is assigned a negative value to mitigate the influence of noise in the guidance image. $I_{\text{size}}$ is the pixel size of the image.

### 2.4 Multi-Focus Image Fusion Based on MUSICA

**Fig. 2:**
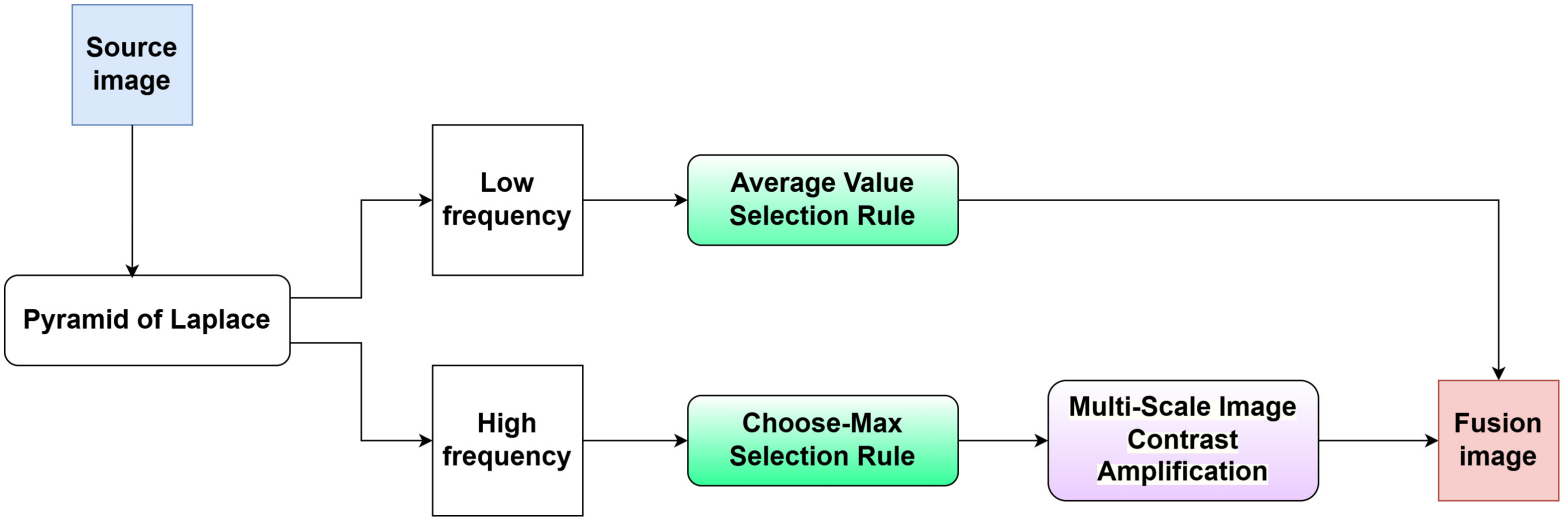
Multi-focus image fusion process.

Defocus blur generated during LSCI imaging inevitably leads to a reduction in image detail in the blurred areas. The high frequencies in an image represent edges and details, so it is necessary to restore the high-frequency details of the blurred parts. Correcting defocus requires the use of images from multiple focal planes. This paper employs a Laplacian pyramid to separate the high- and low-frequency features of a sequence of images and selects the optimal fusion coefficients for each, combining them according to the corresponding coefficients to produce a clear image. Given the insufficient contrast in blood flow velocity images produced by LSCI, amplification of high-frequency signals is necessary. This amplification process is achieved through the use of MUSICA. The process of multi-focus image fusion is shown in Fig. 2.

Low-frequency components, resulting from down-sampling during low-pass filtering, are processed using Gaussian filtering. The contour information in these components can be effectively represented by the average values of multiple datasets, which serve as the fusion coefficient. Conversely, high-frequency components typically employ the choose–max (CM) rule to better emphasize high-frequency details.^33^ The equation for the high-frequency fusion coefficient is

$$c_{F}(m,n)=\{\begin{array}{l} c_{A}(m,n),S_{A}(m,n)\geq S_{B}(m,n)\\ c_{B}(m,n),S_{A}(m,n)<S_{B}(m,n) \end{array},$$ (12)

where $c_{A}(m,n)$ and $c_{B}(m,n)$ are the decomposition coefficients of two images A and B. The coefficient after fusion is denoted as $c_{F}(m,n)$. $S_{A}(m,n)$ and $S_{B}(m,n)$ are measures of the intensity of detail information within the neighborhood centered on (m,n). The decomposition coefficients of high-frequency CM rules may experience irregular changes due to operations such as image translation and rotation, which can amplify the impact of noise. We aim for the selection of intensity in high-frequency detail areas to remain unaffected by interference from any single point. This is achieved by determining the fusion coefficients by selecting the larger absolute values within a 3×3 neighborhood window.

The implementation of MUSICA is based on the Gaussian pyramid, which integrates effectively with the Laplacian pyramid. MUSICA enhances contrast by manipulating the pyramid coefficients, allowing for the nonlinear amplification of small values. This amplification can be expressed as follows:

$$y(x)=aM\frac{x}{\left|x\right|}(\frac{\left|x\right|}{M}{)}^{p},\;-M<x<M,$$ (13)

where x represents the original pyramid coefficient, y(x) is the modified pyramid coefficient, M is the upper limit of the coefficient value, and a is the global amplification factor. The exponent p is used to control the degree of non-linearity.

## 3 Methods and Materials

### 3.1 Animal Experiments

A 25-g adult male C57BL mouse was placed in the experimental area and anesthetized using 2% isoflurane in a 70% $N_{2}O/30\%$ $O_{2}$ mixture. Following anesthesia, the fur on the head was shaved, and an incision was made at the nape of the neck to expose the skull. The animal study was performed in accordance with ethics protocols approved by the Wuhan Myhalic Biotechnological Committee. The experiment utilized a standard circular disk to calibrate the equipment, employing a 785-nm wavelength laser as the light source directed at the vascular region of the mouse’s head. A CCD camera was positioned ∼12  cm above the experimental area to capture images, with a frame rate of 50 Hz and an exposure time of 10 ms. The image resolution was set to 2048×2048  pixels size. Although both the imaging equipment and test subjects were fixated, the respiratory movements of the mice inevitably caused local deformations. Consequently, it was necessary to collect images over a time series to address these changes. So, this work recorded a continuous sequence of 200 raw speckle images. Furthermore, the focal plane was manually adjusted, and 200 frames were captured consecutively at three different focal planes.

### 3.2 Data Analysis

#### 3.2.1 Image registration experiments

From a sequence of 200 continuously captured speckle images, 50 consecutive images were selected for registration. The region of interest was observed with a resolution of 352×352. The first frame was used as the reference image, whereas the remaining images were treated as floating images to be registered. The coordinate transformation model for registration was the TPS model, with no registration and affine transformation models serving as controls. The maximum number of Shi–Tomasi corner detections was set to 500. Due to the speckle characteristics, the original speckle images were relatively blurred, and matching points were concentrated near the vascular structures. A 5×5 convolution kernel was applied to each pixel of the original speckle image to calculate spatial variance. After histogram equalization processing, the vascular structures became clearer.

#### 3.2.2 Image denoising experiments

After image registration, a noisy speckle contrast image is obtained. Various denoising algorithms were then applied to process this noisy image, including ADF, non-local means (NLM), the MD-ABM3D algorithm from Cheng’s study,^17^ and the algorithm introduced in the present work. After performing temporal averaging on the collected 200 image frames, we selected a region of interest (ROI). The resolution of the resulting tLSCI image was 352×352  pixels, which is regarded as the reference. Considering the stability of the denoising effect of guided filtering, the selected matching block size is 8, the search step is half of the block size, and the parameter $\lambda_{\mathrm{TV}}$ in total variation regularization is set to −7. Other parameters remain consistent with those in the original BM3D.

#### 3.2.3 Multi-focus image fusion experiments

In the experiment, image data from multiple focal planes were obtained by manually adjusting the camera focus, and the experimental subject is positioned ∼120  mm from the lens. Images were continuously captured from three distinct focal planes, with 50 frames of speckle images collected for each plane. The image was captured at a distance of 90 mm from the lens. These images were then subjected to registration and denoising processes to generate blood flow velocity images. A multi-focus image fusion algorithm was subsequently applied to these three blood flow images to correct defocus, resulting in sharply focused images. Two regions of interest were selected for detailed observation. Subsequently, two regions of interest were selected for magnified observation. Further analysis was conducted on the blood flow velocity values within these two regions of interest. A rectangular area measuring 50×10  pixels was extracted from each region, and the blood flow velocity values of the 10 pixels in the longitudinal direction were calculated using a weighted average. This served as the reference value for blood flow velocity in that pixel area. The blood flow velocity values of these 50 consecutive pixels were then tabulated for further analysis.

### 3.3 Image Quality Assessment

1. Peak signal-to-noise ratio (PSNR)
  $$\mathrm{PSNR}=10\;\mathrm{lg}(\frac{{\mathrm{MAX}}^{2}}{\frac{1}{mn}\overset{m-1}{\underset{i=0}{\sum }}\overset{n-1}{\underset{j=0}{\sum }}[I-K{]}^{2}}),$$ (14)
  where I is the reference image; K is the image to be evaluated; m and n are the numbers of pixels in the row and column directions of the images, respectively; and MAX is the maximum pixel value of the image. In experiments, the images are typically 8-bit and is usually set to 255. PSNR serves as a reference metric for assessing image quality in terms of the maximum signal value relative to background noise, measured in decibels. A higher PSNR value indicates less distortion in the image.
2. Mean structural similarity index (MSSIM)
  $$\mathrm{MSSIM}(x,y)=\frac{1}{M}\overset{M}{\underset{i=1}{\sum }}\frac{\left(2\mu_{xi}\mu_{yi}+C_{1})(2\sigma_{xi,yi}+C_{2}\right)}{\left(\mu_{xi}^{2}+\mu_{yi}^{2}+C_{1})(\sigma_{xi}^{2}+\sigma_{yi}^{2}+C_{2}\right)},$$ (15)
  where $\mu_{xi}$ and $\mu_{yi}$ are the mean of the image, $\sigma_{xi}$ and $\sigma_{yi}$ are the standard deviation of the image, M is the number of local blocks divided by a certain window size, and $C_{1}$ and $C_{2}$ are the parameters for stabilizing the weak denominator in division operations, typically set to $(0.01\;\mathrm{MAX}{)}^{2}$ and $(0.03\;\mathrm{MAX}{)}^{2}$, which are usually 6.5025 and 58.5225. The value of MSSIM ranges from −1 to 1, from the worst to the best case scenario.^34^
3. Gradient magnitude similarity deviation (GMSD)
  $$\mathrm{GMSD}=\sqrt{\frac{1}{N}\overset{N}{\underset{i=1}{\sum }}(\frac{2m_{r}(i)m_{d}(i)+c}{m_{r}^{2}(i)+m_{d}^{2}(i)+c}-\mathrm{GMSM}{)}^{2}},$$ (16)
  where GMS(i) is the local gradient field, $m_{r}$ and $m_{d}$ respectively denote the gradient magnitudes in the horizontal and vertical directions of the image, and GMSM is the average gradient magnitude similarity, which is obtained by applying average pooling to the GMS map. The value of GMSD ranges from 0 to 1, where a larger value indicates a greater disparity between the test image and the reference image.^35^

## 4 Results and Discussion

### 4.1 Results of Image Registration

**Fig. 3:**
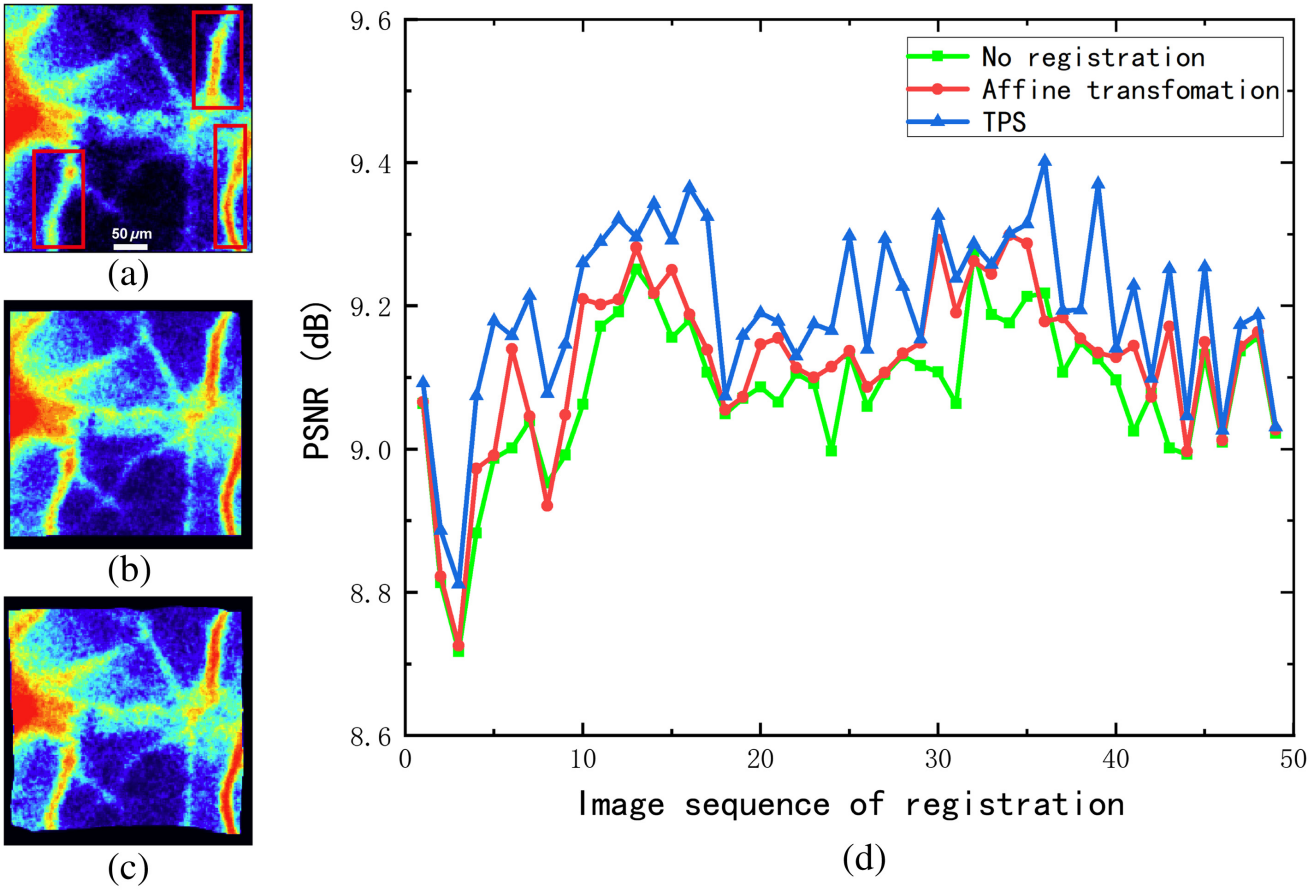
Results of coordinate transformation registration. (a) Blood flow index without registration. (b) Blood flow index obtained after affine transformation registration. (c) Blood flow map obtained after TPS transformation. (d) Result of the PSNR value between multi-frame floating image and reference image under different coordinate mapping.

The results of coordinate transformation registration are shown in Fig. 3. Figures 3(a)–3(c) present blood flow velocity images following various registration processes applied to the speckle images. The unregistered blood flow images show blurry and discontinuous vessels with noticeable gaps. After applying the affine transformation for image registration, the primary vessels become more distinct compared with the unregistered images. However, smaller vessels remain partially blurred. In contrast, using the TPS transformation model for registration reveals more intricate vessel branches in various directions, achieving significantly enhanced imaging quality compared with both prior methods. Figure 3(d) presents the PSNR values comparing each fluctuating image to the reference image. A higher PSNR value indicates greater similarity to the reference image, thereby signifying superior registration quality. Because the signal-to-noise ratio of the blood flow image and preprocessed speckle image is lower than that of the ordinary image, the PSNR is typically lower even after registration. Throughout the continuous registration of 50 frames, the unregistered images exhibit the lowest PSNR values, averaging 9.08 dB, primarily due to motion artifacts that degrade image quality. Upon applying the affine transformation model for registration, there is a slight improvement in PSNR, with an average value of 9.12 dB. However, this model primarily addresses rigid displacement and is insufficient for capturing localized small-range movements inherent in the subject’s motion. In contrast, the TPS transformation model, which accommodates non-rigid displacement, yields a significant enhancement in PSNR, achieving an average of 9.19 dB.

The results indicate that the LK algorithm effectively suppresses inter-frame motion artifacts, but intra-frame motion artifacts (additional blurring of speckle patterns caused by applied motion during the exposure time) remain unresolved, leading to an overestimation of calculated blood flow velocity. Future work could consider integrating the optical flow pyramid algorithm with sub-pixel motion compensation to estimate high-frequency motion trajectories at the sub-pixel level. By splitting the exposure time of a single frame into multiple virtual sub-frames and using optical flow to estimate the displacement between each sub-frame, speckle patterns can be reconstructed. This approach could be combined with a newly proposed linear regression model, incorporating intra-frame velocity as an additional parameter to establish a more accurate blood flow velocity model for correcting motion artifacts.^36^ Optical flow is not only used for image registration but also provides key input parameters for theoretical predictions of motion artifacts through the estimation of two-dimensional surface displacement velocities. By analyzing pixel movement frame by frame, a spatiotemporally continuous two-dimensional velocity field is generated. Combining this with the optical Doppler model, it can predict the speckle contrast reduction caused by the translational motion of each pixel.^37^

### 4.2 Results of Image Denoising

**Fig. 4:**
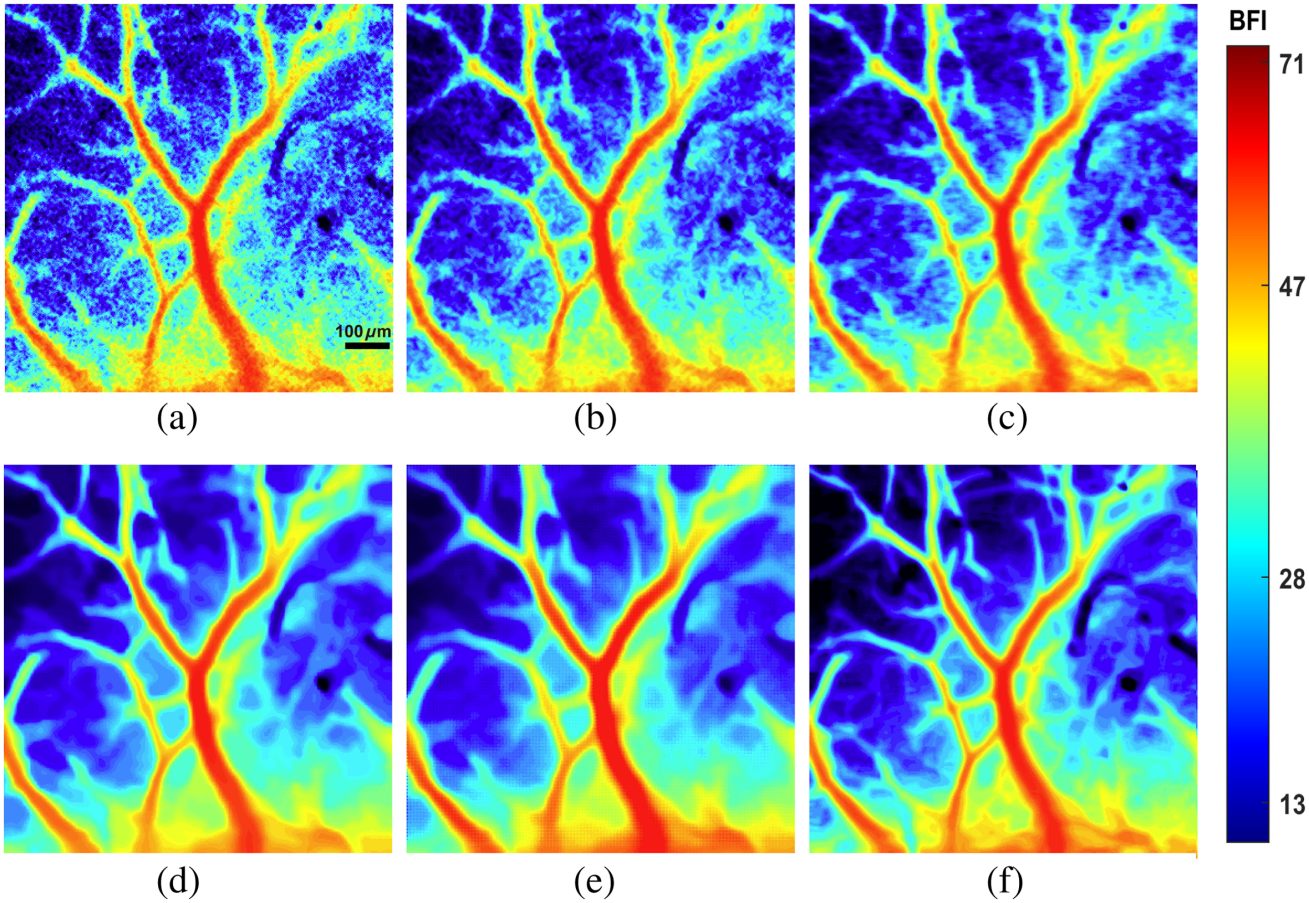
Blood flow index corresponding to different denoising methods. (a) tLSCI. (b) ADF. (c) NLM. (D) MD-ABM3D. (e) Improved BM3D. (f) Reference image.

Finally, blood flow estimation was conducted on the denoised contrast image, and the results as shown in Fig. 4.

Figure 4(a) is the original tLSCI image, which exhibits noise both in the blood vessels and the background. Although tLSCI has made some progress in temporal resolution, there is a significant loss in spatial resolution, resulting in a noticeable decline in overall image quality. The denoising algorithms shown in Figs. 4(b)–4(d) partially reduce non-uniform noise; however, they fail to sufficiently smooth the edges of the blood vessels. In contrast, the improved BM3D method used in this study, when compared with MD-ABM3D, effectively removes noise while preserving the vascular structures to a greater extent, as demonstrated in Fig. 4(e). In addition to using PSNR for denoised image evaluation, the MSSIM and GMSD are also incorporated. In Table 1, which presents the evaluation of image quality. The proposed algorithm demonstrates superior performance compared with other denoising algorithms, as evidenced by higher PSNR and MSSIM metrics and the lowest GMSD metric. A lower GMSD value signifies a smaller discrepancy between the test image and the reference image. Specifically, the PSNR value of the proposed algorithm shows an improvement of nearly 6 dB over tLSCI, whereas the MSSIM value nearly doubles. These results highlight the algorithm’s effectiveness in noise reduction and detail preservation.

**Table 1:** Image quality evaluation table corresponding to each algorithm.

Method	PSNR (dB)	MSSIM	GMSD
tLSCI	21.57	0.48	0.191
ADF	23.79	0.68	0.149
NLM	24.75	0.77	0.126
MD-ABM3D	25.71	0.83	0.110
Improved BM3D	27.52	0.93	0.041

The denoising algorithm proposed in this study refines the MD-BM3D algorithm by integrating guided filtering with total variation regularization. This enhancement mitigates the problem of image detail degradation associated with the Wiener filtering employed during the final estimation stage of BM3D. The proposed method effectively adapts to the noise distribution in speckle images, thus preserving the texture information in the denoised outputs.

### 4.3 Results of Multi-Focus Image Fusion

In the experiment, image data from multiple focal planes were obtained by manually adjusting the camera focus. Images were continuously captured from three distinct focal planes, with 50 frames of speckle images collected for each plane. These images were then subjected to registration and denoising processes to generate blood flow velocity images. A multi-focus image fusion algorithm was subsequently applied to these three blood flow images to correct defocus, resulting in sharply focused images. Two regions of interest were selected for detailed observation. Subsequently, two regions of interest were selected for magnified observation. Further analysis was conducted on the blood flow velocity values within these two regions of interest. A rectangular area measuring 50×10  pixels was extracted from each region, and the blood flow velocity values of the 10 pixels in the longitudinal direction were calculated using a weighted average. This served as the reference value for blood flow velocity in that pixel area. The blood flow velocity values of these 50 consecutive pixels were then tabulated for further analysis. To assess the imaging clarity of different images, this paper employs a no-reference image evaluation standard: sum of modulus of gray difference squared (SMD2). Under this evaluation standard, the larger the value of SMD2, the richer the high-frequency components, indicating a clearer image.^38^

**Fig. 5:**
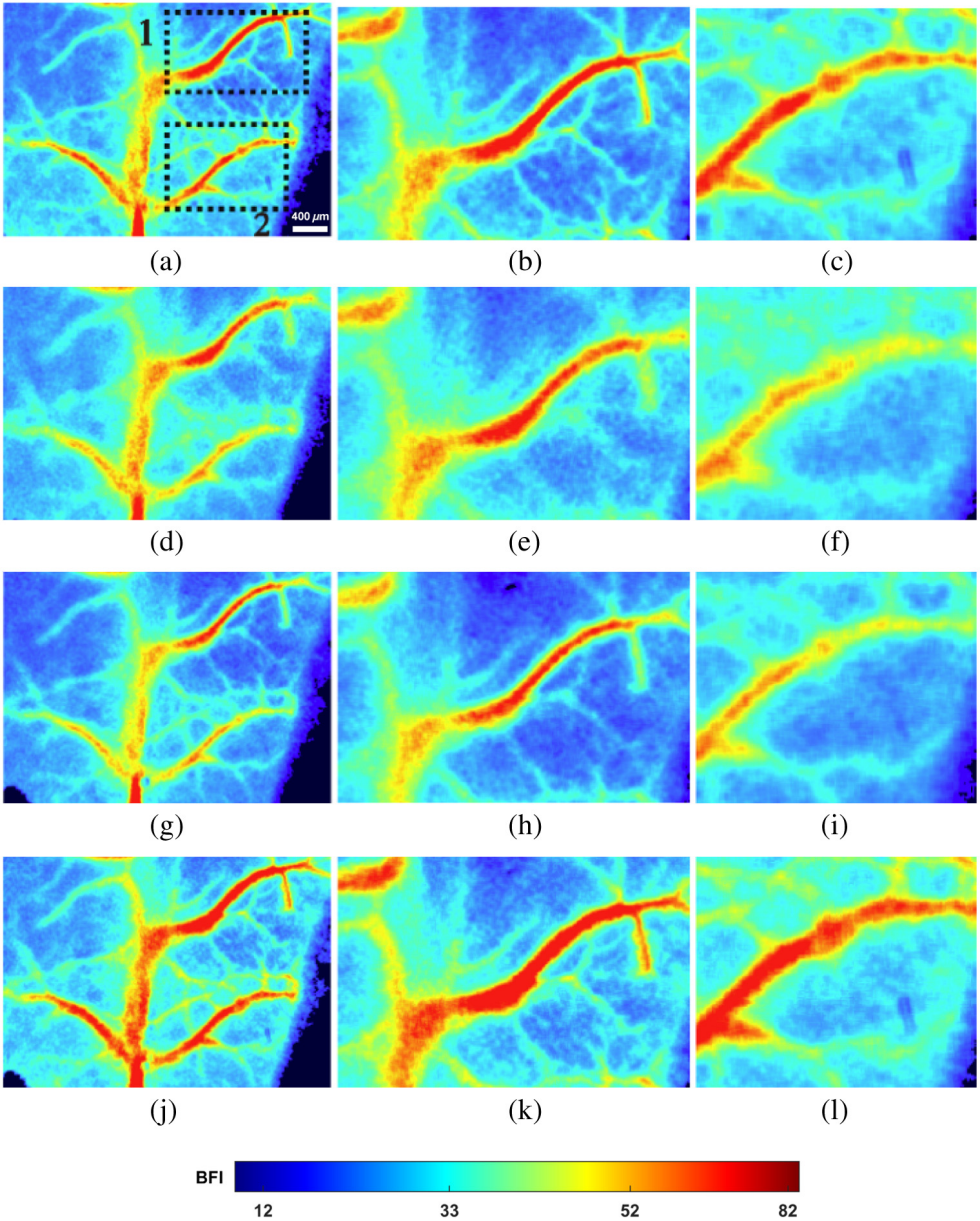
Results of original multi-focus image fusion. (a), (d), and (g) Blood flow images focused on different focal planes. (j) Fused image. (b), (c), (e), (f), (h), (i), (k), and (l) ROI region 1 and 2 images in panels (a), (d), (g), and (j).

In the three sets of images presented in Figs. 5(a), 5(d), and 5(g), it is apparent that both the primary blood vessels and the adjacent smaller vessels exhibit varying levels of blurriness due to differences in focal planes, resulting in a lack of clarity. This issue is particularly pronounced in Fig. 5(d), where the defocusing effect is severe, leading to weak blood flow visibility in the main vessel and more noticeable discontinuities. Following the application of multi-focus image fusion in Fig. 5(j), the morphology of the main vessel becomes more defined, and its trajectory is distinctly visible. However, the smaller blood vessels remain inadequately processed, likely due to insufficient contrast.

**Fig. 6:**
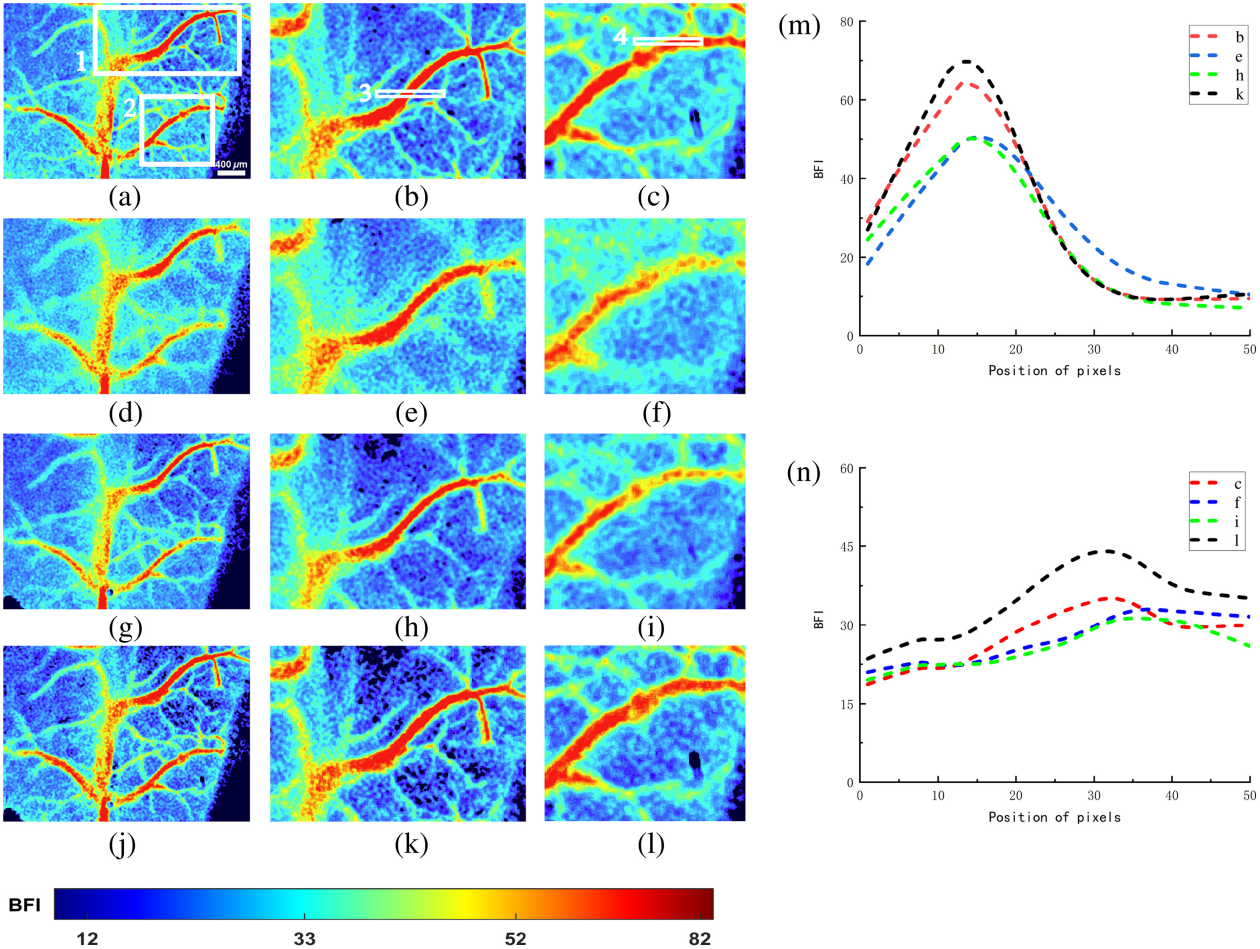
Results and analysis of multi-focus image fusion processed by MUSICA. (a), (d), and (g) Blood flow images focused on different focal planes after MUSICA processing. (j) Fused images based on MUSICA. (b), (c), (e), (f), (h), (i), (k), and (l) ROI region 1 and 2 images in panels (a), (d), (g), and (j). (m) and (n) Blood flow velocity values at pixel positions corresponding to ROI regions 3 and 4 in panels (b), (e), (h), and (k) and (c), (f), (i), and (l).

MUSICA enhances detail signals by restoring high-frequency information. In contrast to Figs. 5(a), 5(d), and 5(g) and Figs. 6(a), 5(d), and 5(g), the primary blood vessels are more prominently highlighted, and the details of smaller blood vessels are effectively restored, resulting in enhanced overall clarity. The defocusing effect is virtually absent in the fused images. Analyzing the blood flow velocity values in Figs. 6(m) and 6(n), the variations in blood flow over 50 pixels in the horizontal direction indicate discrepancies attributable to defocusing, which leads to deviations in blood flow velocity values and subsequently lower final blood flow calculations. Through multi-focus image fusion processing, contributions from multiple focal planes rectify this deviation, restoring the calculated blood flow velocity to standard levels. Furthermore, the foreground and background in the images are more distinctly discernible.

The entire area of the selected image and two regions of interest were processed using the original multifocal image fusion technique. The three focal planes and the final fused image are collectively referred to as Misfocus. From the data in Table 2, it can be observed that the SMD2 values of the defocused images at the three focal planes are relatively low. The two selected regions of interest, due to their different locations, exhibit slight numerical differences. After the multifocal image fusion, the SMD2 values nearly doubled. This indicates that the focus of the fused image has become clearer. The MUSICA algorithm was applied to enhance high-frequency details, followed by multi-focus image fusion processing. The three focal planes and the final fused image are uniformly named Misfocus_MU. The SMD2 values are presented in Table 3, the SMD2 values of the images from each ROI of the three focal planes were three times higher compared with the images that did not use the MUSICA method. The SMD2 value of the multi-focus image fusion enhanced by the MUSICA method was three times greater than the original multi-focus image fusion without the MUSICA method and six times higher compared with the defocused images that did not use the MUSICA algorithm. Furthermore, images processed using MUSICA demonstrated a 50% improvement in clarity compared with defocused images.

**Table 2:** Evaluation table of SMD2 for original multi-focus image fusion.

Focus field	SMD2		
	Full	ROI_1	ROI_2
Misfocus_a	3.493	3.947	4.917
Misfocus_b	2.542	2.912	2.388
Misfocus_c	2.707	3.345	3.098
Misfocus_d	5.674	6.176	6.813

**Table 3:** Evaluation table of SMD2 for multi-focus image fusion with the MUSICA algorithm.

Focus field	SMD2		
	Full	ROI_1	ROI_2
Misfocus_MU_a	13.029	14.086	14.278
Misfocus_MU_b	11.046	11.923	10.992
Misfocus_MU_c	12.515	13.896	14.097
Misfocus_MU_d	21.887	23.376	22.841

Therefore, from the perspective of overall blood flow velocity values and parameter evaluation, the addition of MUSICA to multifocal imaging can effectively improve many cases with poor focus and significantly enhance the image contrast. These enhancements contribute to increased stability in imaging during LSCI experiments.

## 5 Conclusion

This article addresses three important factors affecting vessel visualization in LSCI: motion artifacts, image noise, and defocus effects. First, motion artifacts were eliminated by registering speckle images using the LK optical flow pyramid algorithm, combined with the TPS coordinate transformation model. Then, based on the noise type of the speckle, the BM3D algorithm with total variation regularized guided filtering was applied to LSCI, which removed the noise interference while preserving the fine details of the vessel edges in the blood flow images. These two methods improved the signal-to-noise ratio and resolution of the images. Finally, to address defocus effects, a multi-focus image fusion algorithm based on MUSICA was proposed, incorporating multi-scale image contrast enhancement in the decomposed high-frequency information. This approach produced clear, focused LSCI vessel images, with better contrast between vessels and the background compared with the original multi-focus image fusion effect. This paper improves image quality in three aspects that affect LSCI vessel and blood flow visualization, resulting in better final vessel visualization.

## Data Availability

The code and data that support the findings of this study are available from the corresponding author, A.K.D., upon reasonable request.
